# Surprisal, the PDC, and the primary locus of processing difficulty in relative clauses

**DOI:** 10.3389/fpsyg.2013.00229

**Published:** 2013-05-14

**Authors:** Roger Levy, Edward Gibson

**Affiliations:** ^1^Department of Linguistics, University of CaliforniaSan Diego, San Diego, CA, USA; ^2^Department of Brain and Cognitive Sciences, Massachusetts Institute of TechnologyCambridge, MA, USA; ^3^Department of Linguistics and Philosophy, Massachusetts Institute of TechnologyCambridge, MA, USA

Of the ambitious purview of MacDonald's (2013) article, we find the part fleshed out in most concrete detail—the comprehension consequences of her Production-Distribution-Comprehension (PDC) theory, the easiest to comment upon. Such a theory as she has sketched out would be extraordinarily compelling: a theory that, in contrast with accounts relying on “innate parsing biases,” posits that “comprehension results reflect distributional regularities in the language” that “comprehenders are generating expectations for upcoming input,” places “emphasis on the role of learning probabilistic constraints,” makes use of “extensive language corpora” to “[permit] comprehension researchers to examine the relationship between production patterns … and comprehension behavior” and thereby “reframes our understanding of sentence comprehension.” The only way we can see such a theory being more compelling would be for it to be specified precisely enough to be computationally implementable and to make *quantitative* and *localized* predictions about the processing difficulty of every word in a sentence that could be tested rigorously on a variety of linguistic materials. A naïve reader of MacDonald's article may not know that such a theory already exists and has been highly successful. This theory, known as *surprisal*, was first proposed by Hale (2001), building on early ideas by Attneave (1959) from the dawn of information theory (Shannon, 1948) and cognitive science.

As proposed by Hale (2001) and elaborated by Levy (2008), surprisal theory posits that comprehenders use fine-grained probabilistic knowledge derived from linguistic experience to form expectations both about the structural interpretation of what has already been encountered in the input and about what input may yet be upcoming, and that these expectations immediately determine processing difficulty (with a precise, quantitative difficulty metric) and guide interpretation preferences. The theory has been applied to a variety of languages and linguistic phenomena, it has been tested on comprehension behavior of both specific grammatical constructions (e.g., Brouwer et al., 2010; Levy et al., 2012) and naturalistic datasets (Boston et al., 2008; Demberg and Keller, 2008), and the functional form of its incremental difficulty metric has been empirically confirmed (Smith and Levy, 2008, 2013).

In the first empirical case discussed by MacDonald, surprisal theory predicts the local interpretation preference for precisely the reasons articulated by MacDonald for her PDC theory. Moreover, the Distribution-Comprehension (DC) part of MacDonald's theory—the idea that the empirical distribution of various syntactic and semantic properties of language determines probabilistic (hence defeasible) processing preferences—is explicit in models predating surprisal, including not only the constraint-based approaches she mentions but also in the probabilistic parsing approach of Jurafsky (1996); and since distribution can only be derived from production, it seems to us that the Production-Distribution (PD) part is implicit. The theoretical advance of surprisal over these earlier probabilistic and constraint-based approaches is very specific: it unified probabilistic resolution of structural ambiguity already present in the input with the formation of expectations regarding future input. It is unclear what corresponding conceptual advance is provided by MacDonald's account.

This brings us to the second empirical case of comprehension behavior discussed by MacDonald: the processing of relative clauses (RCs). We deeply appreciate the point that the relative production frequencies of subject and object RCs are highly sensitive to a variety of factors including (but not limited to) language, NP type (e.g., pronominal vs. full; Reali and Christiansen, 2007), and agent and patient animacy (e.g., Gennari et al., 2012). We also agree that an impressive body of research points to the generalization that the comparative processing difficulty of subject vs. object RCs is often well-predicted by their relative frequencies given these factors (Traxler et al., 2002; Reali and Christiansen, 2007; Gennari and MacDonald, 2008; Levy et al., 2013; cf. Fedorenko et al., 2011; Gibson et al., in press; and see also Doyle and Levy, 2010; Gibson and Wu, 2013 for counter-examples). Nevertheless, we do not believe that any extant theory in the class MacDonald proposes adequately explains all the critical facts in the syntactic complexity of relative clauses. In particular, the critical data bear not only on which types of RCs are hardest to comprehend, but also on the *locus* of maximal processing difficulty. This point is extremely clear for the classic SRC/ORC processing difficulty differential for English:

(1a) The reporter that attacked the senator admitted the error. (SRC)
(1b) The reporter that the senator attacked admitted the error. (ORC)

A surfeit of theories—both experience- and memory-based—correctly predict that the ORC *that the senator attacked* in (1b) is harder than the SRC *that attacked the senator* in (1a). However, fully incremental experience-based theories such as surprisal fail to correctly predict *where* difficulty arises in (1b). As pointed out by Hale (2001), Grodner and Gibson (2005), and Levy (2008), experience-based theories predict the locus of processing difficulty for ORCs to be at the onset of the RC noun phrase *the senator*, which in (1b) disconfirms the comprehender's rational expectation that the RC will turn out to be subject-extracted. Although recent work (Staub, 2010) has revealed some degree of processing difficulty at this point in ORCs like (1b), the bulk of the difficulty clearly falls on the RC verb *attacked*. This difficulty occurs despite the fact that the RC verb in ORCs should be more expected, not less expected, than in SRCs: there are fewer syntactic events that can occur after *The reporter that the senator …* than after *The reporter that* …, and there are fewer things that a senator can do to a reporter than that a reporter can do (see discussion in Grodner and Gibson, 2005 and Levy, 2008). These are precisely the conditions under which surprisal correctly predicts difficulty differentials for a variety of languages and constructions (Konieczny, 2000; Vasishth and Lewis, 2006; Nakatani and Gibson, 2008; Levy and Keller, 2013), yet surprisal fails in the case of English RCs (and it seems to fail similarly for Russian RCs; Levy et al., 2013). Crucially, this problem for surprisal and similar experience-based theories arises regardless of whether one considers the preceding context, *The reporter that…*, to be structurally “ambiguous”: as MacDonald suggests, it is clear that there is considerable indeterminacy as to how the sentence will continue at this point, including indeterminacy as to the grammatical role of the head noun. The key point is that where most of this indeterminacy is pruned away—at the RC noun phrase onset—is not where the differential difficulty is largest^1^.

We consider theories of syntactic processing making reference to explicit, costly (and/or potentially fallible) memory operations, such as those of Gibson (1998, 2000) and Lewis and colleagues (2005, 2006), of continued importance in the study of RC comprehension because they make the right predictions not only about *what* is difficult but about *where* the difficulty is observed in this heavily studied empirical domain.

Although MacDonald's proposal in its present form has not made theoretical commitments as precise as those of surprisal, it is not clear how her proposal could be cashed out to make precise predictions about *where* processing difficulty occurs in a way that avoids the same empirical difficulties that surprisal runs into. This is not to say that there is no hope for developing purely experience-based theories of processing difficulty that explain currently problematic data such as those we describe above. But we do not believe that any such theory currently exists, and we are not sure how to develop one ourselves.

## Footnotes

^1^Hale (2003, 2006) presents an alternative experience-based theory of incremental processing difficulty, the Entropy Reduction Hypothesis, that purportedly predicts the SRC/ORC processing difficulty differential with success. However, we do not believe that this proposal is ultimately empirical viable (see e.g., discussion in Levy et al., 2013).
